# Interfacial Crystallization and Supramolecular Self-Assembly of Spider Silk Inspired Protein at the Water-Air Interface

**DOI:** 10.3390/ma14154239

**Published:** 2021-07-29

**Authors:** Pezhman Mohammadi, Fabian Zemke, Wolfgang Wagermaier, Markus B. Linder

**Affiliations:** 1 VTT Technical Research Centre of Finland Ltd., FI-02044 Espoo, Finland; 2 Department of Biomaterials, Max Planck Institute of Colloids and Interfaces, 14476 Potsdam, Germany; f.zemke@tu-berlin.de (F.Z.); wolfgang.wagermaier@mpikg.mpg.de (W.W.); 3 Department of Bioproducts and Biosystems, School of Chemical Engineering, Aalto University, FI-02150 Espoo, Finland; markus.linder@aalto.fi

**Keywords:** spider silk, X-ray diffraction, β-sheet, skin formation, coacervate, liquid–liquid phase separation, conformational conversion

## Abstract

Macromolecular assembly into complex morphologies and architectural shapes is an area of fundamental research and technological innovation. In this work, we investigate the self-assembly process of recombinantly produced protein inspired by spider silk (spidroin). To elucidate the first steps of the assembly process, we examined highly concentrated and viscous pendant droplets of this protein in air. We show how the protein self-assembles and crystallizes at the water–air interface into a relatively thick and highly elastic skin. Using time-resolved in situ synchrotron x-ray scattering measurements during the drying process, we showed that the skin evolved to contain a high β-sheet amount over time. We also found that β-sheet formation strongly depended on protein concentration and relative humidity. These had a strong influence not only on the amount, but also on the ordering of these structures during the β-sheet formation process. We also showed how the skin around pendant droplets can serve as a reservoir for attaining liquid–liquid phase separation and coacervation from the dilute protein solution. Essentially, this study shows a new assembly route which could be optimized for the synthesis of new materials from a dilute protein solution and determine the properties of the final products.

## 1. Introduction

Fabricating materials from biological macromolecules includes utilizing unique molecular interactions to formulate condensed multiscale superstructures. The formation of protein-based materials is closely related to multi-scale supramolecular self-assembly processes and conformational conversion by developing secondary structural motifs, which is not yet fully understood [1,2]. One fascinating example of such ultrastructural material is spider silk, which exhibits exceptional mechanical properties in comparison to any natural or human-made materials. It is a unique material, with high stiffness, strength, and extensibility, and considerable overall toughness [3,4].

Despite extensive study in recent years, and the fact that spider silk and the spinning process have served as a source of inspiration for the design of next-generation high-performance materials, little is known about the intermediate process steps from dilute spidroin solution to the final dried silk fiber, nor the conformational conversions taking place during this process at ambient conditions [5,6,7,8,9,10]. Riekel and Vollrath investigated dragline spider silk strand extracted from living spiders in an in situ X-ray experiment by combining small-angle X-ray scattering (SAXS) and wide-angle X-ray scattering (WAXS) measurements [11]. They showed that the fibers are composed of crystalline and amorphous domains, stabilized by a polymeric network. β-sheet structures were present even shortly after exiting the spider, demonstrating that the secondary structures form either directly at the exit or already inside the body [12]. There is a general understanding that the remarkable mechanical properties of spider silk are heavily influenced by β-sheets motifs [6,13,14,15,16].

Although this has been mainly investigated for dried silk solutions or fibers, little is known about the formation processes that occur in the protein solutions while drying, or the development of secondary structural features. Several synthetic pathways exist to produce recombinant silk, and they provide an excellent basis for in situ investigations of the formation process of secondary structures [17].

**Figure 1:**
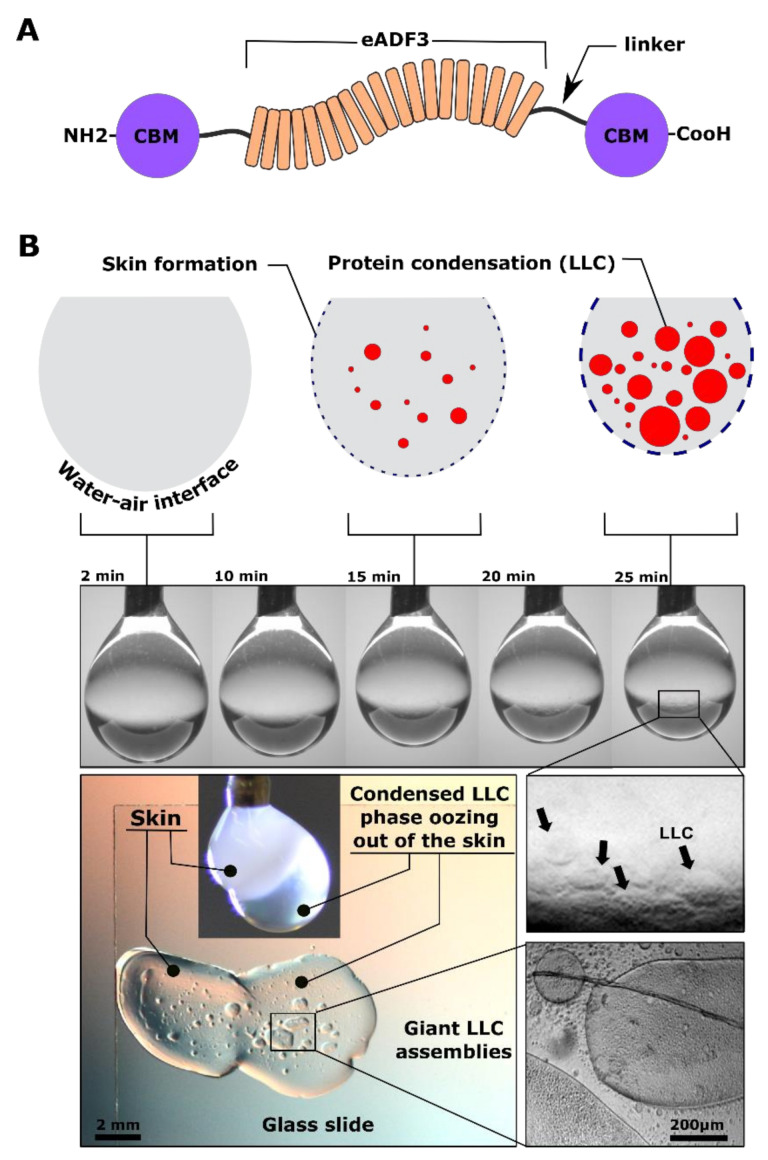
Structural evolution during evaporation induced self-assembly of pendant droplet of the silk-like protein. (**A**) Schematic representation of the genetically engineered and recombinantly produced spider silk-like 3-block architecture. (**B**) This results in two distinct assemblies. A highly crystalline skin at the water–air interface and liquid–liquid phase separation (coacervate formation) internally.

In this work, we explore the development of conformational structures during evaporation-induced self-assembly of pendant droplets of recombinantly produced spider silk-like proteins (Figure 1A,B). Their tri-block architecture is based on our earlier reports consisting of two folded terminal domains and a highly repetitive amphiphilic mid-block spidroin sequence (Figure 1A) [18,19,20,21]. We anticipated the geometry of the pendant droplet interface to be the most suitable approach for understanding the self-assembly and conformational evolution of dense silk solution with concentrations of spidroins in the same range as in the glands before fiber formation (Figure 1B). This is otherwise problematic to probe using any other means, such as planar surfaces using very dilute protein solutions, as described previously [22,23]. As a starting point, under controlled relative humidity at room temperature, we formed a pendant droplet in the air from a never-dried 2% *w*/*v* protein in pure water for one hour. To our surprise, we noted that the same protein could undergo two very distinct self-assemblies. First, part of the protein is self-assembled and crystallized at the water–air interface into an elastic skin (Figure 1). Second, we observed that the skin acts as a barrier, enabling the protein within to gradually be concentrated and undergo liquid–liquid phase separation (LLPS) and thereby forming liquid-like coacervate (LLC) droplets (Figure 1). We extensively characterized the physio-chemical nature of the LLPS for this protein previously [18,19,20,21]. Therefore, in this work we focused on a detailed characterization of self-assembly, leading to the formation of the skin with the associated evolution of secondary structures.

## 2. Materials and Methods

### 2.1. Cloning, Expression and Protein Purification

Cloning, expression, and protein purification were performed as described in our earlier publications [18,19,20,21]. Briefly, DNA sequences encoding bacterial family three cellulose-binding module (CBM3) from *Ruminiclostridium thermocellum* (PDB Accession number: 1NBC) [24] and DNA sequence encoding Aaraneus diadematus major ampulla gland (ADF3) and twelve-time repeats of residues 325–368 (eADF3) [25,26,27] were synthesized and codon-optimized by GeneArt gene synthesis (ThermoFisher Scientific, Waltham, MA, USA) for expression in *E. coli*. The construct was made by golden gate cloning [28,29,30]. pE-28a (+) (kanR) (Novagen, Darmstadt, Germany) expression vector in frame with C-terminal 6×His-tag coding sequence for facilitating the purification was used. The resulting construct was named CBM-eADF3-CBM, in which the repetitive silk sequence was flank at both terminals with CBM. In general, for all the expressions either EnPresso^®^ B medium (BioSilta, Oulu, Finland) technology were used or MagicMediaTM *E. coli* expression medium (ThermoFisher Scientific, Waltham, MA, USA) according to instructions, with some changes otherwise stated in the text. After 15–24 h of induction, cells were harvested (centrifugation at 16,000× *g*, 15 min, 4 °C), washed, and lysed. Proteins were purified using HisTrap FF crude columns (GE Healthcare life Science, Chicago, IL, USA) connected to AKTA-Pure liquid chromatography. For high scale purification cells lysed and supernatant heat-shocked at 70–75 °C for 30 min, and then centrifuged at 16,000× *g* for 80 min at 4 °C and desalted using Econo-Pac^®^ 10DG column (Bio-Rad Laboratories, Hercules, CA, USA). Samples were then frozen in liquid nitrogen and stored at −80 °C until use.

### 2.2. Sample Preparation for the Analysis

Never-dried protein samples were gradually concentrated to the desired concentrations (0.2% *w*/*v*, 2% *w*/*v*, and 15% *w*/*v*) using centrifugal concentrator (Vivospin20, Göttingen, Germany) at 845 r.c.f. Unless otherwise stated, proteins were in Milli-Q water. For the measurements, pendant droplet of silk solution made by forming approximately 200–500 µL of proteins solution drawn into a Hamilton^®^ syringe and a droplet with approximately 40 µL volume was made in a humidity-controlled box using a flat-tipped needle at various relative humidities (20%, 40%, and 80% relative humidity (RH)). Self-assembly of proteins at the air–water interface led to the formation of an elastic skin around the pendant droplets.

### 2.3. Optical and Polarized Microscopy

Samples were imaged on several platforms as follows: (1) Axio observer inverted microscope (Carl Zeiss, Jena, Germany) equipped with a motorized stage, AxioCam MRm camera (Zeiss, Jena, Germany), a × 100/numerical aperture, and Zeiss AxioVision software (version 4.7). Images were further processed with ImageJ [31] or ImageJ Fiji (versions 1.47d) [32]. (2) Polarized microscopy imaging was performed using a Leica DM4500 P LED polarized optical microscope (POM, Wetzlar, Germany) for the qualitative observation and to study the birefringence of the fibers. Samples were placed between two cross-polarizers in an optical microscope and interference color resulting from the retardation between ordinary and extraordinary waves at angles ±45 was determined. All the imaging was performed on microscope glass slides (24 mm × 50 mm, with 170 µm thickness) that were washed with 10 mM NaOH with sonication for 5 min and rinsed with water.

### 2.4. Flash-Freezing of the Pended-Droplet for Characterization of the Skin Formation

Verification of the pended droplets was achieved by plunging the droplets into 1:1 mixture of liquid propane and ethane (−180 °C). Samples were then handled under liquid nitrogen transferred into a FreeZone 4.5 L Cascade Benchtop Freeze Dry Systems equipped with a collector cooling chamber of −105 °C.

### 2.5. Scanning Electron Microscopy (SEM)

High-resolution SEM imaging was performed via Zeiss (Sigma VP) FE-SEM field emission microscope (Microscopy Center, Aalto University, Espoo, Finland) with variable pressure, operating at 1–1.5 kV operating. A thin (2 nm) platinum coating was sputtered onto the samples prior to imaging of surfaces only in SEM mode. For further analysis, an image processing software package ImageJ [31] and ImageJ Fiji (versions 1.47d) [32] was used.

### 2.6. Raman Spectroscopy

A Lab RAM HR UV-NIR single-stage dispersive Raman microscope system with an 800 mm high-resolution spectrograph was used to record Raman Spectra from the specimens. A 633 nm @ 30 mW (with an estimated power of 3 mW on the sample) Helium-neon laser was used to focus on the surface of the specimens. An Olympus BX41 with a motorized stage with 50× objective was used to focus the <1 μm laser beam to collect the scattered radiation and record the spectra with a high-resolution CCD camera (1024 × 256 (pixel size 26 μm × 26 μm)) in the region of 700–1800 cm^−1^ with the exposure time of 20 s, accumulation number of 100 and grating of 600.

### 2.7. Micro-Mechanical Measurement of Skin

Tensile testing was performed on a 2 kN tensile/compression module (Kammrath & Weiss GmbH, Schwerte, Germany) using 2 N load cells. The elongation speed was 8.35 µm/s with a gauge length of 10 mm. The ends of the fiber-shaped materials were fixed by gluing them between two pieces of abrasive sandpaper. Before testing, the samples were stabilized at 50% relative humidity overnight. SEM imaging was used to obtain the cross-sectional areas of each sample. In most cases, the cross-section was not completely circular; a contour line was drawn to measure the cross-sections. We used the Image J software package.

### 2.8. Interfacial Tension

Measurements were performed using Biolin Scientific Attention Theta Flex optical tensiometer (Espoo, Finland) equipped with a temperature and humidity controlled unit with the camera resolution of 1984 px × 1264 px (3009 FPS). A pendant drop was made using a micropipette containing 25 μL of either 0.2%, 2%, or 15% *w*/*v* silk solution. The Young−Laplace formula was used to calculate the shape change of the pendant drop [33,34]. The pendant drops were incubated at room temperature in a controlled humidity unit. Changes in the shape of the pendant droplet were characterized using OneAttention Theta Flex software (version 3.8).

### 2.9. Synchrotron Wide-Angle X-ray Scattering (WAXS) Measurement

The in situ X-ray scattering experiments were conducted at BESSY II (Helmholz Zentrum Berlin für Materialien und Energie; Berlin, Germany) at the µSpot beamline (Max Planck Institute of Colloids and Interfaces, Potsdam, Germany). An energy of 15 keV with a Si111 double monochromator and 100 µm spot-size was used; the sample to detector distance of 311 mm allowed for WAXS investigations. The images were captured with an Eiger 9M detector (pixel size 75 µm × 75 µm). To control the humidity, a custom-made measurement chamber (10 to 90% relative humidity) was installed, which was attached to a generator and its air pressure system. This chamber consists of X-ray transparent polymeric windows and a cannula (2 mm diameter) that could be operated remotely with a motorized tensile testing machine. The defrosted proteinous solutions (0.2%, 2%, and 15% *w*/*v* silk solution) were captured inside the cannula, and a droplet was formed. A repeating line scan (4 mm width, step size 200 µm, and 10 s exposure time) was used to acquire images directly underneath the cannula over the whole width of the droplet; each sample was investigated for a minimum of 1 h with higher durations for the high humidities. The software DPDAK (directly programmable data analysis kit) was used for calibration and evaluation of the X-ray spectra, using the plugins “Image Input” and “Image Integration 1D” [35]. A quartz standard was used for calibration of the radially integrated images, oversaturated pixels were removed by masking and the background of the empty measurement chamber was subtracted. Furthermore, a transmission correction value was calculated for each measurement point, considering the respective path of the beam through a spherical shape. Finally, Lorentzian peak fits (5000 iterations, least-squares, linear background subtraction) were applied to determine the area and position of the peaks; peak position variations higher than 0.5 and error calculations higher than 0.1 were discarded.

## 3. Results and Discussions

### 3.1. Evolution of Skin at the Water–Air Interface

**Figure 2:**
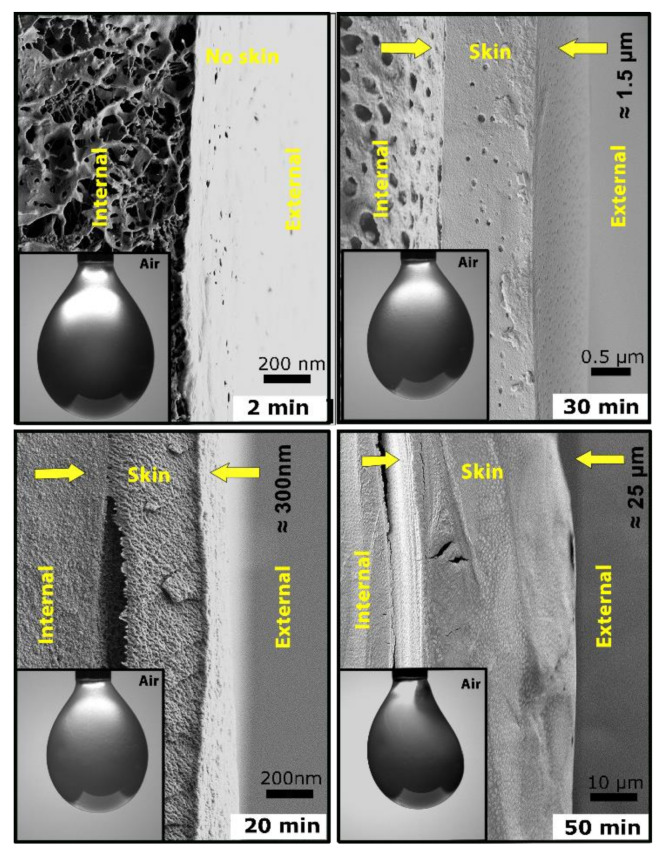
Self-assembly and evolution of skin at the water–air interface. SEM images of the cross-section of the pendant droplets of 2% *w*/*v* silk solution after 2 min, 20 min, 30 min, and 50 min. The skin layer is indicated in-between two yellow arrows for all images. For each time point, we have also included representative images of each pendant droplet.

To better understand the assembly process of the protein at the interface, we vitrified the entire droplets at various time points in liquid ethane–propane (50%:50%) mixture, followed by fracturing the droplets and probing the evolution of the skin using high-resolution SEM imaging to have direct observation of the assembly. Figure 2 illustrates droplets corresponding to 2 min, 20 min, 30 min, and 50 min into the incubation in air, respectively. The formation of a free-standing coherent skin, covering the entire surface of the pendant drop over time, is clearly evident. The thickness of the skin increased as more protein was absorbed at the interface of the drop. The thickness of the skin ranged from 10 nm to about 25 µm during incubation from 2 to 50 min. Notably, the skin appeared to become increasingly dense as it matured.

### 3.2. Structural Evolution of Skin

**Figure 3:**
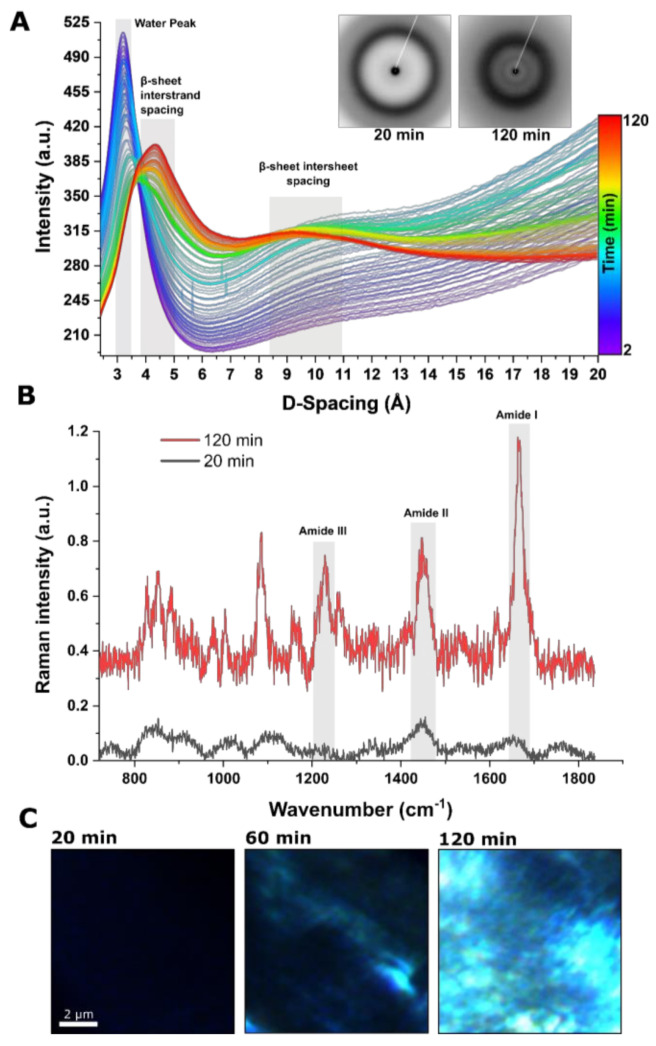
Probing structural evolution of skin at the water–air interface using time-resolved in situ synchrotron WAXS measurements corresponding to Figure 2. (**A**) Extracted one-dimensional diffractogram for the period of 120 min. (**B**) RAMAN spectrum for the first and last time points as in (**A**). (**C**) Polarized microscopy images of skins at various time points placed between two crossed polarizers at 45° with respect to their axis, illustrating the increase in the birefringence of the specimens over time.

To investigate the structural features of the skin at the molecular scale, we followed in situ self-assembly of the protein at the water–air interface using synchrotron WAXS measurements. Figure 3A shows representative time-resolved 1D WAXS curves extracted from 2D diffraction patterns corresponding to Figure 2. The measurement was expanded up to 120 min. Scans were performed every minute at 500 µm underneath the blunt needle tip at the center point, keeping the position fixed throughout the measurement. At the onset of the measurement, a strong signal corresponding to the so-called water peak could be seen with the D-spacing ranging from 3 to 3.5 Å [36,37]. Over time, scattering intensity declined due to the gradual evaporation of the water molecules. This gradually resulted in the emergence of the two visible peaks towards the last scans. A sharp peak between 4 and 5 Å, with the maximum peak intensity at around 4.5 Å, is known to correspond to β-sheet interstrand spacing, as described previously, by combining experimental and computational simulation [19,38,39,40], A broader peak appeared between 8.5 and 11 Å, with its maximum intensity at 10.6 Å. This spacing is commonly assigned to β-sheet interspacing. As the content of β-sheet structures was rising during the drying process, water loss and drying can be interpreted as the key triggering mechanism for crystallization [18,38,39,40]. These observations are in agreement with complementary RAMAN spectroscopy measurements to determine the conformationally sensitive amide I signal which has been dominantly assigned to β-sheet structures with an emerging peak at a wavenumber of 1657 cm^−1^ (Figure 3B) [41,42]. Additionally, performing polarized microscopy of the skin at various time points placed between two crossed polarizers at 45° with respect to their axis demonstrated an increase in the birefringence of the samples over time, indicative of conformational conversion and evolution of β-sheet motifs (Figure 3C).

### 3.3. Mechanical Properties of the Skin

**Figure 4:**
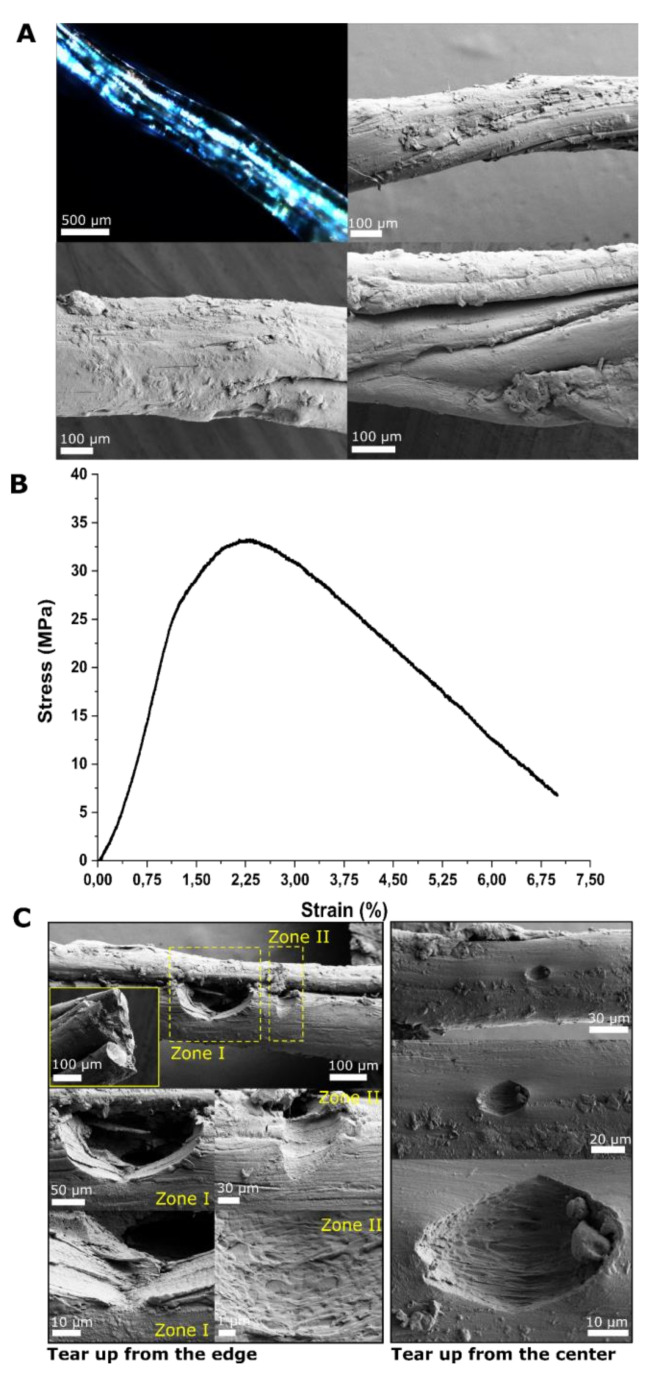
Rolling the skin into fiber-shaped material and testing its mechanical properties. (**A**) Polarized microscopy of a fiber-like material made from the skin. The panel also shows SEM images from three different regions of the same fiber which demonstrates inhomogeneity in the thickness and surface morphology throughout the length of the fiber. (**B**) Representative stress–strength curve for the corresponding sample as in (**A**). (**C**) SEM images of the material after the tensile measurement test illustrating typical tears observed throughout the length of the fibers.

As the skins matured over time into the free-standing, highly elastic 2D membrane in both the hydrated and dehydrated state, we set to determine their mechanical properties. To minimize possible experimental errors during tensile measurements due to inherent variation in the thickness of the skin throughout the surface of a single droplet, and also possible variation between different droplets, we rolled the 2D skins into fiber-shaped materials (Figure 4A). This enabled us to measure the cross-sectional area for each specimen accurately by SEM. By doing so, we were able to make fiber-shaped materials with an average diameter of 500 ± 30 μm. Figure 4A,B shows the results of stress–strain tests carried out for the corresponding samples at 50% RH. As described, the skins were highly elastic in the hydrated state, however their ultimate strength values were substantially low, and therefore we were not able to detect this using our experimental setup. Hence, we exclude any discussion on the hydrated specimens. However, for the dried samples at 50% RH, the general shape of the stress–strain curves showed ductile fracture features [43]. Stress–strain curves of the skins showed four distinct regimes: (1) a linear elastic deformation region from 0 to 1.12% strain and 0 to 25 MPa stress, (2) a yield point (1.24), (3) a short hardening region expanding from 1.12 to 2.6% strain and 25 to 33.2 Mpa stress, followed by (4) decreasing plastic deformation region until catastrophic failure. The materials showed an ultimate strength of 33.2 ± 4.6 MPa and an ultimate strain of 6.89%. High-resolution fractography after the tensile test revealed the origin of the ductile behavior of the materials. Besides the internal molecular rearrangement and sliding of the protein backbone, we noted the formation of multiscale tears throughout the material, which is indicative of inelastic deformation and a decrease in the tensile properties before undergoing catastrophic failure.

### 3.4. Absorption Kinetics of the Protein at the Water–Air Interface

**Figure 5:**
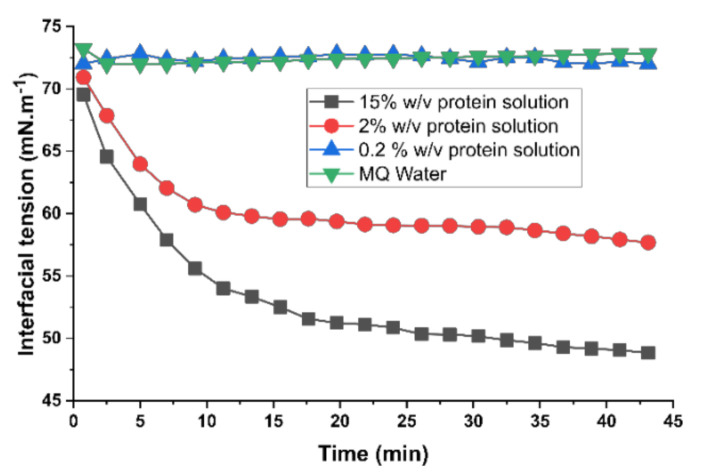
Changes in the interfacial tension of silk solution during drying over time.

To better understand the absorption kinetics of the protein at the water–air interface, we performed pendant drop tensiometry for the protein solutions at the various concentrations (Figure 5). In addition to what we indicated as a saturated silk solution (2% *w*/*v*), we also tested very dilute (0.2% *w*/*v*) and highly concentrated (15% *w*/*v*) solutions. We also included MQ water as a control. Data obtained from the DST measurements revealed two distinct trends illustrating the absorption of the protein at the water–air interface, very similar to what is known for surface-active proteins [44,45]. First, with the increase in protein concentration, we noted a substantial decrease in surface tension. Second, surface tension decreased substantially as the droplets were incubated for a longer period. These were only pronounced for 2% *w*/*v* and 15% *w*/*v*. DST data points for the 0.2% *w*/*v* sample remained nearly constant at around 72.5 mN m^−1^, indicating that there is no apparent absorption of the proteins at the interface. Data for 0.2% *w*/*v* was nearly identical to the MQ water. Both 2% *w*/*v* and 15% *w*/*v* showed a similar trend in the evolution of the interfacial tension. This includes a steep initial decrease in the surface tension, followed by a very gradual reduction for the rest of the measurements. However, we also observed a few noticeable differences. For instance, surface tension for the 2% *w*/*v* decreased from 71 to 58 mN m^−1^, whereas this was ranging from 69 to 47 mN m^−1^ for the 15% *w*/*v* protein solution. Additionally, the initial plunge was expanded for around 10 min for the 2% *w*/*v*, whereas this was about 20 min for 15% *w*/*v* sample.

### 3.5. Time-Dependent Conformational Conversion of the Proteins at the Water–Air Interface

**Figure 6:**
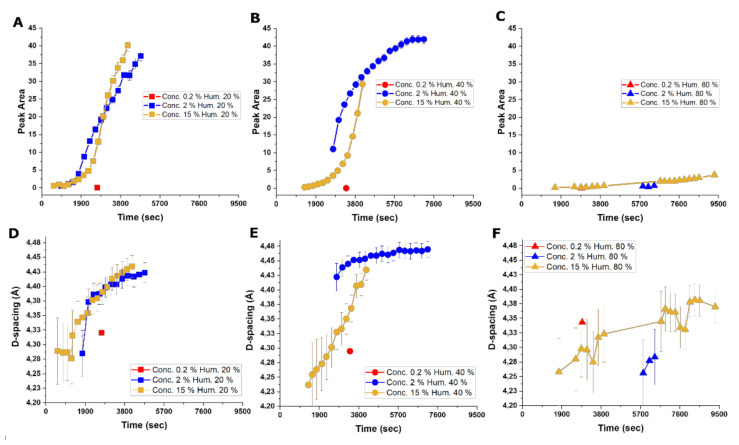
Effect of concentration and humidity on the formation of β-sheets analyzed by in situ X-ray scattering. (**A**) Peak fit area over time for protein concentrations of 0.2% *w*/*v*, 2% *w*/*v*, and 15% *w*/*v* protein samples at 20% RH, (**B**) at 40% RH, and (**C**) at 80% RH. (**D**) Peak fit position illustrating changes in D-spacing (shown in angstrom scale) as a function of time for protein concentrations of 0.2% *w*/*v*, 2% *w*/*v*, and 15% *w*/*v* protein samples at 20% RH, (**E**) at 40% RH, and (**F**) at 80% RH.

To approach the structural characteristic and conformational conversion more systematically we probed in situ X-ray scattering experiments on drying droplets (Figure S1, Supplementary Materials) of 0.2%, 2%, and 15% *w*/*v* at various humidities (20, 40, and 80% RH). We primarily examined the most prominent peak at 4.5 Å to evaluate the formation of secondary structures. The area and the position of this peak were probed as a function of time (drying of the droplets) and the circular chord (corresponding to the distance the X-ray beam has to travel through the droplet). Measurements were performed at various positions of the droplets, starting from the edge and moving to the center (Figure 6A–F and Figure S2). Results demonstrated that the formation of β-sheet structures was directly correlated with the increase in the protein concentration and inversely correlated with the increase in humidity. We noted that the amount of β-sheets, indicated by the peak area (Figure 6A–F and Figure S2), was lowest for 80% RH and protein concentrations of 0.2% *w*/*v*, reaching almost the range of a threshold error.

Samples with 2% and 15% *w*/*v* at 20 and 40% RH showed a clear appearance of β-sheet structures over time. Both 2% and 15% *w*/*v* samples showed relatively similar development of β-sheets over time, with the differences that samples with 15% *w/v* reach a higher content of β-sheets in comparison to 2% *w*/*v* in most humidities. However, it is notable that the 15% *w*/*v* sample showed a lower slope (Figure 6A–C) of the peak area, increasing overtime at the onset of measurements. Seidel et al. showed that a peak position at around 4.4 Å is correlated to silk with only isolated or partially stacked β-sheet structures, while materials (usually after drawing) with larger microcrystals exhibit a peak for lateral distance at 4.6 Å. Therefore, not only larger peak areas, but also higher peak positions (larger D-spacing) can be assumed to correlate with a higher degree of ordering of β-sheets [38]. In general, D-spacing rises overtime for all samples (Figure 6D–F).

It is also important to notice that, in almost all the combinations, a slight delay before β-sheet formation was observed. This was more pronounced for low protein concentrations and high humidities. Contrary to this, the combination of high protein concentrations and low humidities showed the least delay, leading to the assumption that the water content and the drying of the droplet is of great importance for the entire process. Furthermore, looking at the β-sheet formation over the circular chord (corresponding to the distance the X-ray beam has to travel through the droplet), for most samples, we noted that the assembly is higher at the edge of the droplet, but we found no relation between the path through the droplet and the peak position, and therefore ordering, of the β-sheets.

## 4. Conclusions

Natural and synthetic spider silk materials are both mechanically processed to achieve their mechanical properties, usually by a spinning process. Earlier studies showed that the three-dimensional poly(alanine) crystals with the β-sheet structure are formed only during drawing steps [38]. Future studies may cover both the early formation stages of secondary structures, as well as their transformation into higher-order structures due to additional processing. Another phenomenon in natural spider silk, which strongly depends on the basic formation mechanisms of secondary structures, is the so-called super contraction, where unrestrained spider dragline silk contracts to about 50% of its original length when wetted or exposed to highly humid environments [46]. Our results also show that a humidity of 80% leads to a significant reduction in β-sheets. Specifically, our results provide insights into the formation processes of secondary structures from solutions containing proteins inspired by spidroins. This work thereby highlights the importance of the water–air interface during the self-assembly of tough silk fiber in the natural spinning process, and clarifies why synthetic processes, i.e., wet spinning of engineered or reconstituent silk proteins, do not recapitulate the properties of native fibers. On a more general level, these results build the basis for further investigations of such formation processes to optimize the production of new materials with tailored functionalities from solutions of engineered macromolecules toward sustainable production of next-generation high-performance material with much broader applications.

## Data Availability

Data sharing is not applicable for this article.

## Supplementary Materials

The following are available online at https://www.mdpi.com/article/10.3390/ma14154239/s1, Figure S1: Overview of the used humidity measurement chamber with a motorized tensile testing machine, syringe/cannula, and X-ray transparent polymeric windows. The red circle marks the area of interest, where the droplet was formed; Figure S2: Effect of concentration and humidity on the formation of β-sheets analyzed by in-situ X-ray scattering. (A) Peak area fit over the circular chord for 0.2% *w*/*v*, 2% *w*/*v* and 15% *w*/*v* protein samples at 20% RH, 40% RH and 80% RH. (B) Peak fit position over the circular chord of the droplet corresponding to (A). For better clarity, data points for 80% RH for all three concentrations were removed due to the unreliability of detections.
