# Supplementary material for: Interfacial Crystallization and Supramolecular Self-Assembly of Spider Silk Inspired Protein at the Water-Air Interface
Source: Materials (Basel). 2021 Jul 29;14(15):4239. doi: 10.3390/ma14154239 (PMC8348448; doi:10.3390/ma14154239)
Supplement: Supplementary file 1 [file materials-14-04239-s001.zip › materials-1312504-supplementary.pdf]

materials

Supplementary Materials

Interfacial Crystallization and Supramolecular Self-Assembly of Spider Silk Inspired Protein at the Water-Air Interface

Pezhman Mohammadi [1,*], Fabian Zemke [2,†], Wolfgang Wagermaier [2] and Markus B. Linder [3]

1 VTT Technical Research Centre of Finland Ltd., FI-02044 Espoo, Finland
2 Department of Biomaterials, Max Planck Institute of Colloids and Interfaces, D-14476 Potsdam, Germany; f.zemke@tu-berlin.de (F.Z.); wolfgang.wagermaier@mpikg.mpg.de (W.W.)
3 Department of Bioproducts and Biosystems, School of Chemical Engineering, Aalto University, FI-02150 Espoo, Finland; markus.linder@aalto.fi
* Correspondence: Pezhman.mohammadi@vtt.fi
† Current address: Fachgebiet Keramische WerkstoffeChair of Advanced Ceramic Materials, Institute of Materials Science and Technology, Technische Universität Berlin, 10623 Berlin, Germany.

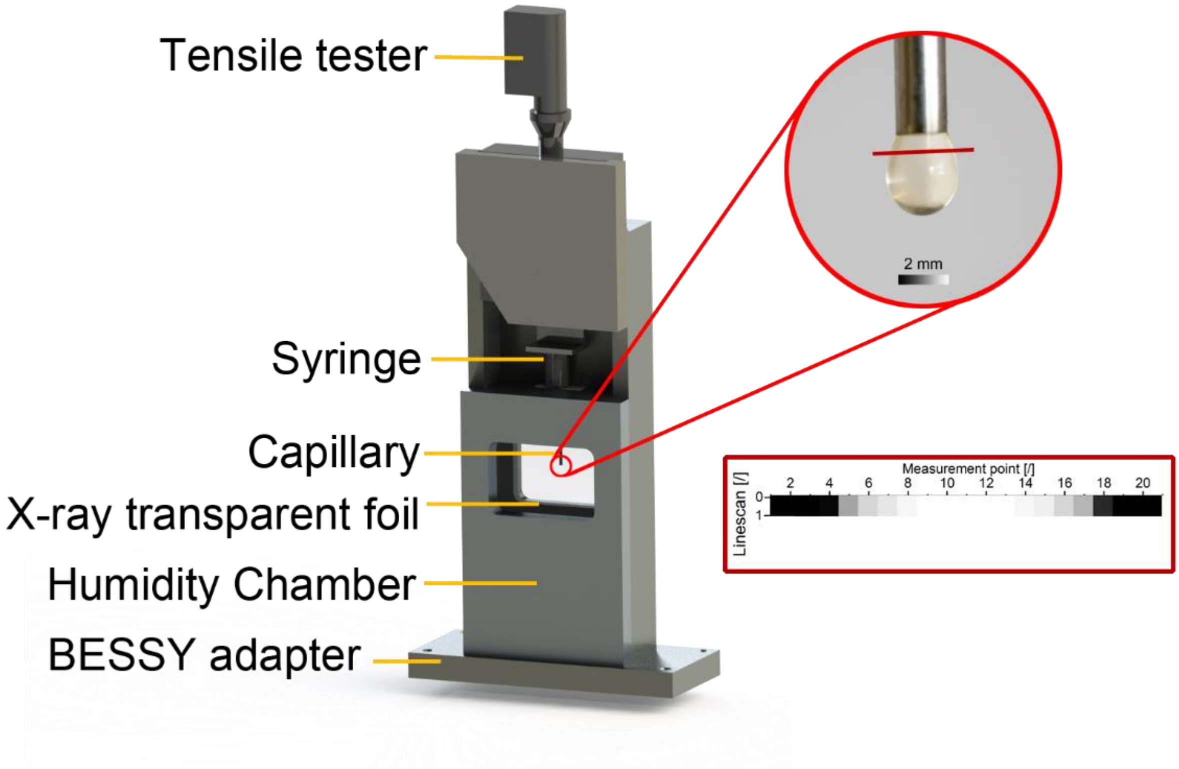

Figure S1. Overview of the used humidity measurement chamber with a motorized tensile testing machine, syringe/cannula, and x-ray transparent polymeric windows. The red circle marks the area of interest, where the droplet was formed.

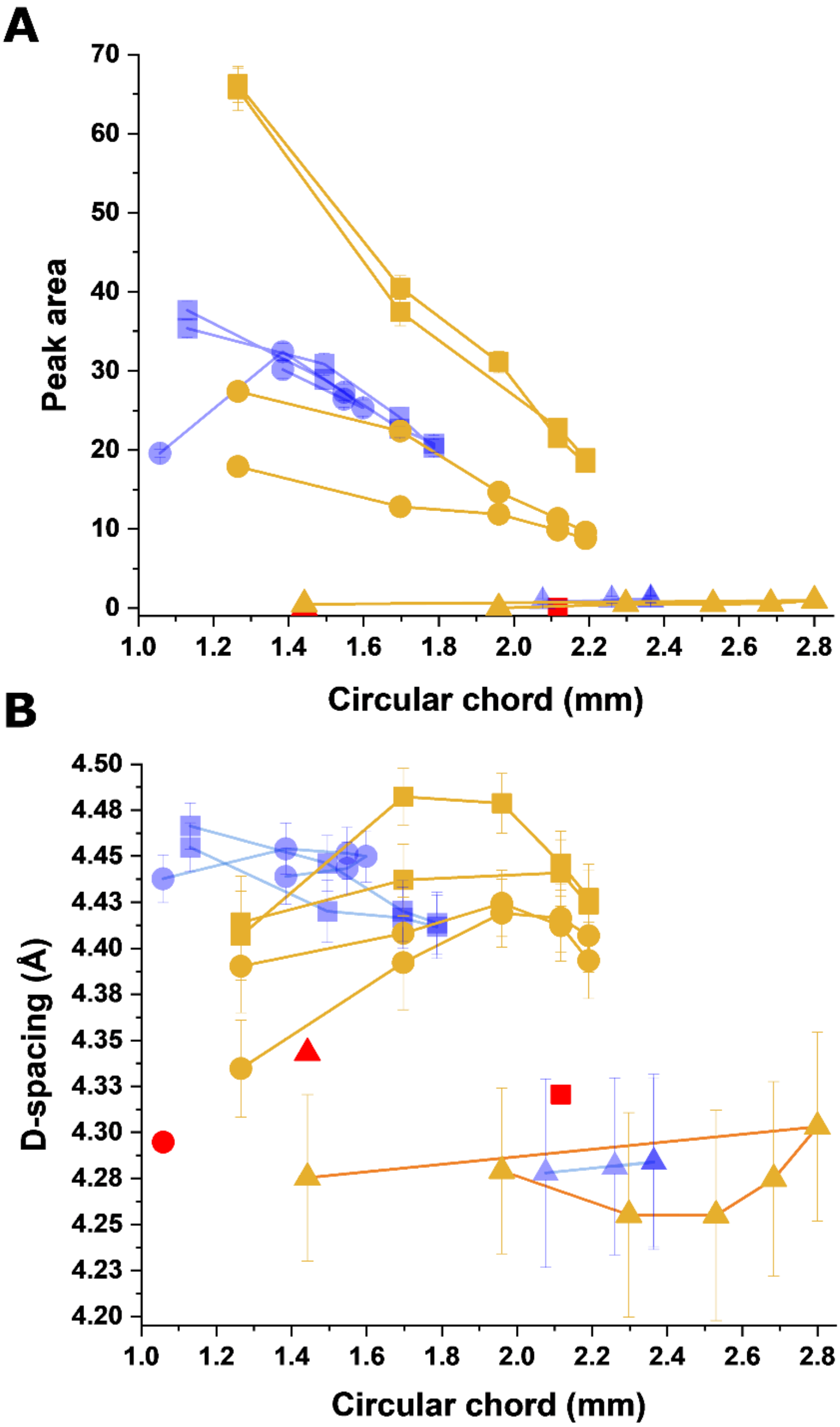

Figure S2. Effect of concentration and humidity on the formation of β-sheets analyzed by *in-situ* x-ray scattering. (**A**) Peak area fit over the circular chord for 0.2% w/v, 2% w/v and 15% w/v protein samples at 20% RH, 40% RH and 80% RH. (**B**) Peak fit position over the circular chord of the droplet corresponding to A. For better clarity, data points for 80% RH for all three concentrations were removed due to the unreliability of detections.
